# Modeling direction discrimination thresholds for yaw rotations around an earth-vertical axis for arbitrary motion profiles

**DOI:** 10.1007/s00221-012-3120-x

**Published:** 2012-05-24

**Authors:** Florian Soyka, Paolo Robuffo Giordano, Michael Barnett-Cowan, Heinrich H. Bülthoff

**Affiliations:** 1 Department of Human Perception, Cognition and Action, Max Planck Institute for Biological Cybernetics, Spemannstraße 38, 72076 Tübingen, Germany; 2 Department of Psychology, The Brain and Mind Institute, Natural Sciences Centre, Western University, Ontario, N6A 5B7 Canada; 3 Department of Brain and Cognitive Engineering, Korea University, Anamdong, Seongbuk-gu, Seoul, 136-713 Korea

**Keywords:** Vestibular, Psychophysics, Semi-circular canals, Threshold, Model, Self-motion, Transfer functions, Rotation

## Abstract

Understanding the dynamics of vestibular perception is important, for example, for improving the realism of motion simulation and virtual reality environments or for diagnosing patients suffering from vestibular problems. Previous research has found a dependence of direction discrimination thresholds for rotational motions on the period length (inverse frequency) of a transient (single cycle) sinusoidal acceleration stimulus. However, self-motion is seldom purely sinusoidal, and up to now, no models have been proposed that take into account non-sinusoidal stimuli for rotational motions. In this work, the influence of both the period length and the specific time course of an inertial stimulus is investigated. Thresholds for three acceleration profile shapes (triangular, sinusoidal, and trapezoidal) were measured for three period lengths (0.3, 1.4, and 6.7 s) in ten participants. A two-alternative forced-choice discrimination task was used where participants had to judge if a yaw rotation around an earth-vertical axis was leftward or rightward. The peak velocity of the stimulus was varied, and the threshold was defined as the stimulus yielding 75 % correct answers. In accordance with previous research, thresholds decreased with shortening period length (from ~2 deg/s for 6.7 s to ~0.8 deg/s for 0.3 s). The peak velocity was the determining factor for discrimination: Different profiles with the same period length have similar velocity thresholds. These measurements were used to fit a novel model based on a description of the firing rate of semi-circular canal neurons. In accordance with previous research, the estimates of the model parameters suggest that velocity storage does not influence perceptual thresholds.

## Introduction

The vestibular system, important for our perception of self-motion and our sense of balance, has been intensively studied in animals. The seminal work of Fernandez and Goldberg (1971) with squirrel monkeys significantly advanced the understanding of the response of vestibular afferent neurons to inertial motion stimulation. In humans, perceptual thresholds for sinusoidal accelerations are often measured using motion simulators in order to study the vestibular system. The aim of the present study is twofold: to extend threshold measurements for rotational stimuli to non-sinusoidal accelerations and to explain these threshold measurements with a model based on the firing rate of semi-circular canal neurons.

Threshold measurements for non-sinusoidal accelerations are important for at least two reasons. First, it has not yet been shown whether perceptual thresholds can be described based on a linear system, for example, if thresholds for non-sinusoidal stimuli can be predicted from thresholds for sinusoidal stimuli. Second, self-motion is often non-sinusoidal. Natural head movements, for example, are never purely sinusoidal, even if the motions are trained (Crane and Demer 1997). Furthermore, when using a motion simulator to present stimuli, high-frequency movements can exhibit distortions from the commanded sinusoidal motion. This latter problem will also be discussed in detail in the “Methods” section. Current models of perceptual thresholds do not distinguish between sinusoidal and non-sinusoidal accelerations; rather, they only take into account the period length of the motion. Here, we propose a new model that can take into account both the period length and the specific time course of the acceleration for a given stimulus.

For linear systems, it is sufficient to measure the response to sinusoidal stimuli, because the response to non-sinusoidal periodic stimuli can be predicted from the sinusoidal responses. However, a threshold is an inherently non-linear concept. Therefore, it is non-trivial to predict thresholds for non-sinusoidal motions from thresholds for sinusoidal motions. It is known from the work of Fernandez and Goldberg (1971) that the firing rate of vestibular afferent neurons can be described by a linear system. We propose a model that uses a threshold for the firing rate in order to describe perceptual thresholds. Therefore, our model consists of two parts: a linear part describing the firing rate and a non-linear threshold. The idea of placing a threshold after the firing rate has previously been used in spatial orientation models (Borah et al. 1988). However, it has not been shown yet whether such an approach is able to describe perceptual thresholds for yaw rotations, for example, if perceptual thresholds for arbitrary motion stimuli can be described based on a threshold for the firing rate of vestibular afferent neurons.

There have been a number of attempts to model perceptual thresholds based on the dynamics of the semi-circular canals. For example, Benson et al. (1989) measured the perceptual response to transient (one-period) sinusoidal acceleration stimuli with varying period length (varying frequency^1^). They proposed that the threshold for correctly discriminating the direction of motion should be inversely related to the sensitivity of the vestibular system: The lower the threshold, the more sensitive the system. They found that the threshold depends on the length of the period of the stimulus: The shorter the period, the higher the threshold in terms of peak acceleration.^2^ This behavior is in agreement with the response of vestibular neurons to rotational accelerations (Fernandez and Goldberg 1971): The shorter the period, the lower the gain of a transfer function describing the firing rate. A low gain corresponds to a low sensitivity of the system and therefore to a high perceptual threshold.

Benson et al. (1989) did not fit a dynamic model to their data, but only performed a first-order regression because their range of tested periods was rather small. However, they noted that if a dynamic model was used, the transient of the model should be taken into account. This is due to the fact that the peak response (peak firing rate) to a transient (one-period) sinusoidal acceleration significantly differs from the peak response to a steady-state sinusoidal acceleration. This is especially noticeable for high-frequency stimuli: For instance, by using the transfer function proposed by Grabherr et al. (2008), the peak response to a transient 3-Hz sinusoidal stimulus is about twice as high as the steady-state response.

Grabherr et al. (2008) measured thresholds over a large range of frequencies using the same stimuli as Benson et al. (1989). Since the semi-circular canals exhibit high-pass filter characteristics, Grabherr et al. (2008) fit the steady-state gain of an inverted high-pass filter transfer function to their threshold data. While this allows fitting the data, it does not take into account the transient of the filter response. Another drawback of their model is that only the frequency and not the time course of the stimulus is taken into account.

In a study by Heerspink et al. (2005), thresholds for stimuli consisting of sinusoidal motions with increasing amplitudes were measured over a large range of frequencies. They used an approach proposed by Hosman and van der Vaart (1978) to fit their data. The steady-state gain of a transfer function model describing the dynamics of the firing rate of vestibular neurons (van Egmond et al. 1949; Fernandez and Goldberg 1971) was fit to the inverted threshold measurements. This approach is able to describe their threshold measurements as a function of the stimulus frequency, but it does not take into account the specific time course of the stimulus. The same approach was also used by Valente Pais et al. (2006) to fit thresholds for pitch motions.

The main shortcomings of the models described above are that they are based on the steady-state response of the vestibular system and that they do not take into account the specific time course of the stimulus. Here, a new modeling approach is proposed which takes into account both the transient and the profile shape of the motion stimulus. We previously reported work on translational thresholds using a modeling approach with a transfer function description of the firing rate of otolith neurons (Soyka et al. 2011). In this paper, rotational thresholds are described using a transfer function for the semi-circular canals. Threshold measurements for stimuli varying in frequency as well as in profile shape are presented, and we show that our model can accurately fit these measurements. Since we report thresholds in terms of peak velocity, and the firing rate of semi-circular canal neurons is proportional to stimulus velocity (Fernandez and Goldberg 1971), we expect to find similar thresholds for different profiles with the same frequency.

## Methods

Since the methods (motion stimuli, threshold estimation, modeling) employed in this study are similar to those employed in our previous work, we refer the interested reader to Soyka et al. (2011) for in-depth descriptions.

### Participants

Ten participants (5 females) took part in the study. They were 21–34 years old and reported no vestibular problems. The participants were paid a standard fee. They did not receive any feedback about their performance during the study. The experiment was conducted in accordance with the requirements of the Helsinki Declaration.

### Motion stimuli

**Fig. 1:**
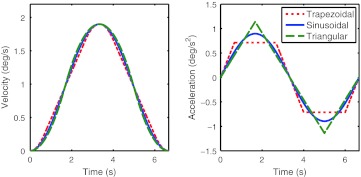
*Left* commanded yaw rotation velocity for the three profile shapes. *Right* corresponding angular acceleration of the profiles. The profiles were named according to the shape of their acceleration. Note that clearly distinct acceleration profiles have quite similar velocity profiles 95 × 48 mm (300 × 300 DPI)

Three different types of motion profiles were used in this study. Each consisted of a head-centered yaw rotation around an earth-vertical axis with period length *T*, while the time course of the accelerating and braking phases differed across types. The profiles were named according to the shape of their accelerations: *trapezoidal*, *sinusoidal*, and *triangular* (Fig. 1). These profile shapes are similar to those of our previous study investigating translational motions. However, here the trapezoidal profile was slightly changed so that the peak acceleration is reached after *dt* = *T*/10 s as opposed to *dt* = *T*/9.91 s as used previously.

The three profiles were tested for three different period lengths *T*: 0.3, 1.4, and 6.7 s, for a total of nine conditions. These periods were chosen because they fall in between periods that were investigated by Grabherr et al. (2008). In order to present these motion stimuli to participants, we used the Max Planck Institute CyberMotion Simulator. Further details on its hardware and software specifications are available (Robocoaster, KUKA Roboter GmbH, Germany; Teufel et al. 2007; Robuffo Giordano et al. 2010a, b; Barnett-Cowan et al. 2012).

**Fig. 2:**
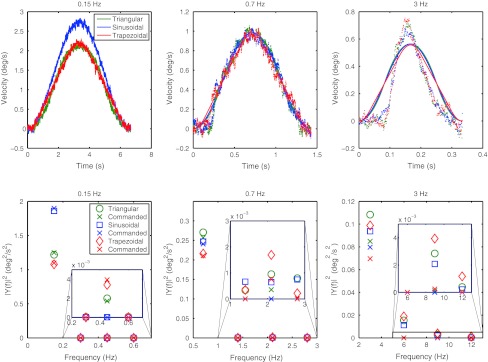
*Upper row* gyroscope measurements (*dots*) at threshold level intensity together with the commanded motions (*solid lines*). *Lower row*: Power spectra of the measured and the commanded signals. The *insets* show a zoom of the 2nd, 3rd, and 4th harmonic. It can be seen that the 3-Hz profiles are not correctly reproduced. Note that this figure is best viewed in color 273 × 193 mm (300 × 300 DPI)

High-frequency motions are difficult to reproduce with any type of simulator. In order to assess the actual motion of the device, a gyroscope (Analog Devices ADXRS150) was attached to the seat of the simulator and yaw velocity was measured at 1,000 Hz. The nine conditions were measured at threshold level intensity and averaged over 40 trials. The averaged measured yaw velocities and their power spectra (Hsu 1995) are plotted in Fig. 2 against the commanded velocities. Although the expected differences between velocity profile shapes are small, it can be seen from both the averaged velocities and the spectra that the profiles are very well reproduced for the 0.15-Hz stimuli. For the 0.7-Hz stimuli, the commanded peak velocities are well reproduced, but the profile shapes exhibit slight distortions. However, the 3-Hz profiles are clearly distorted and the commanded peak velocities are also not correctly reproduced.

The goal of our study was to measure and model thresholds for different profile shapes. For the higher-frequency stimuli, the actual profile shapes deviate from the commanded shapes. However, and most importantly, the measured profiles still differ between conditions (see the spectra in Fig. 2), allowing us to assess the influence of the profile shape on the thresholds. But it has to be noted that, especially for the 3-Hz stimuli, the actual velocities did not exactly follow the commanded profiles. One of the advantages of the proposed modeling approach is that it can deal with arbitrary signal shapes. Therefore, the averaged measured signal shapes at threshold level intensity were used as input for the model in place of the commanded signal shapes, since they more accurately represent the actual motion imposed on our participants.

In addition to the distortions in profile shape, the measured peak velocities of the 3-Hz signals differed from the commanded peak velocities. In order to correct for these differences, additional velocity measurements (40 trials) were performed at the highest and the lowest measured threshold intensities (see Fig. 5) for the 3-Hz conditions. This was done to test whether the needed correction factors depend on the stimulus intensity.

The offsets between the peak of the commanded and the measured velocities were calculated for each trial. Paired sample *t*-tests showed no significant differences between the offsets for the highest and the lowest intensities for each of the three 3-Hz conditions [triangular: *t*(39) = −1.139, *p* = .262; sinusoidal: *t*(39) = 0.584, *p* = .562; trapezoidal: *t*(39) = −0.660, *p* = .513]. Therefore, each measured 3-Hz threshold is corrected by adding the mean offset between the commanded and the measured peak velocities (triangular: 0.244 deg/s; sinusoidal: 0.232 deg/s; trapezoidal: 0.210 deg/s).

### Experimental procedures

A one-interval, two-alternative forced-choice task was used to measure velocity thresholds for direction discrimination of a head-centered yaw rotation around an earth-vertical axis. Participants initiated a trial with a button press, and after a 1-s pause, the movement began. They were rotated either leftward or rightward, were instructed to indicate the direction of their rotation as fast as possible via a button press, and were then moved back to the starting position with the same speed as the stimulus previously delivered. In total, there was at least a 2-s period of standstill between consecutive motions (longer if participants did not immediately start the next trial). The duration of the break might seem too short when comparing it against the time constant for the semi-circular canals of about 5 s (Fernandez and Goldberg 1971). However, the stimuli are around threshold level (around the spontaneous firing rate of the semi-circular canals), and the measured thresholds are comparable to previous studies. Additionally, none of our participants reported any perception of motion aftereffects. Therefore, we believe the duration of the break was sufficient. No feedback about performance was provided.

A within-participants design was employed. In order to counterbalance possible learning effects, the presentation sequence of the conditions was randomized with the constraint that the same profile type was never presented consecutively. The participants were seated in a chair with a 5-point harness and wore light-proof goggles. Acoustic white noise was played back during the movements via headphones. Participants wore clothing with long sleeves and trousers, and a fan was directed toward the participant’s face to mask possible air movement cues during the movement of the simulator. Participants were tested in two sessions of approximately 2.5 h each on two separate days. After a maximum of 15 min, a break was scheduled in order to prevent fatigue.

### Threshold estimation

**Fig. 3:**
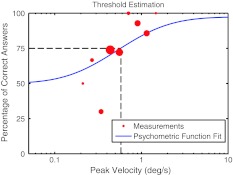
A psychometric function ranging from 50 % chance level to 100 % correct discrimination performance was fit to the data in the logarithmic stimulus space. The discrimination threshold is defined as the peak velocity needed to correctly report the direction of motion 75 % of the time. Since an adaptive sampling method was used, the amount of trials per stimulus intensity varied. The size of each *dot* indicates the number of tested trials 112 × 84 mm (300 × 300 DPI)

During the experiment, the peak velocity of the tested profile was varied in order to measure a psychometric function ranging from 50 % chance level to 100 % correct discrimination performance. The inflection point of the psychometric function is located at 75 %, and thus, the *discrimination threshold* is defined as the peak velocity needed to correctly report the direction of motion 75 % of the time (Fig. 3). Note that previous work incorrectly referred to the same task as direction detection and not direction discrimination (Benson et al. 1989; Grabherr et al. 2008). In general, data for a discrimination task are analyzed by fitting a psychometric function ranging from 0 % rightward answers to 100 % rightward answers. This would allow for an estimate of the point of subjective equality between leftward and rightward motions (the bias) as well as an estimate for the discrimination threshold. Here, we chose to use detection analysis fitting a psychometric function ranging from 50 to 100 % correct answers to be consistent with and comparable to previous literature (Benson et al. 1989; Grabherr et al. 2008). Also note that fitting to the percentage of correct discrimination assumes that there are no differences in discrimination performance between leftward or rightward motions. Benson et al. (1989) showed that on average (30 participants) there were no significant differences in threshold estimates if only leftward or rightward stimuli were evaluated. Although this does not provide a direct measure of asymmetries in performance, it indicates that on average asymmetries can be neglected for threshold estimation. Indeed, post hoc tests of the discrimination performance of individual participants did not reveal any significant differences between leftward and rightward rotations near threshold level. For further details, we refer the reader to the discussion.

The psychometric function was modeled as a cumulative normal distribution in logarithmic stimulus space. The logarithmic spacing was chosen because Benson et al. (1989) showed that the frequency of correct discrimination can be well approximated by a cumulative normal distribution if the stimulus intensity is expressed in logarithmic units. The logarithmic stimulus spacing is further motivated by studies providing evidence that the perception of rotation follows a logarithmic law (Elsner 1971; Clark and Stewart 1972; Mallery et al. 2010). The mean of the underlying normal distribution coincides with the 75 % point. A lapse parameter, ranging between 0 and 5 %, was included into the fit to take into account the possibility of accidentally pressing the wrong button even if the direction was correctly perceived. It has been shown that this can significantly improve the fit (Wichmann and Hill 2001).

A Bayesian adaptive procedure, based on the method proposed by Kontsevich and Tyler (1999), was used to estimate discrimination thresholds (Tanner 2008). This method fits a psychometric function after each newly acquired data point to the whole data set. Simulating the answer of the next trial for each possible acceleration stimulus allows for calculating which stimulus would most change the fit of the psychometric function. This stimulus is considered the most informative and is presented as the next trial. Making use of this method allows for fast and accurate estimation of the threshold.

Twenty stimulus intensities (peak velocities) were used with logarithmic spacing between 0.045 deg/s and 7.2 deg/s for the *T* = 0.3 s profiles, 0.053 deg/s and 8.4 deg/s for the *T* = 1.4 s profiles, and 0.117 deg/s and 13.333 deg/s for the *T* = 6.7 s profiles. After each trial, the Bayesian adaptive method provides an estimate of the threshold and the variance of that estimate. To determine the threshold of a profile, participants were tested until the variance of the estimate was below a previously defined value—*the break value*—for 20 consecutive trials and until at least 80 trials were performed.

In order to choose a suitable break value, preliminary experiments were performed with 3 additional participants. Each participant was tested in 3 conditions (200 trials per condition) without using a stopping criterion but rather a fixed amount of trials. The estimated threshold after 200 trials was regarded as the best possible estimate. The absolute difference AD_trial_ between the best possible estimate and the estimate of the method after each trial was recorded together with the variance of the estimate after each trial. The break value for the variance was chosen such that the absolute difference AD_trial_ was smaller than 10 % of the average threshold (averaging over the profile shapes for a fixed profile period length) for all 9 data sets (AD_trial_ < 0.05 deg/s for 3 Hz, <0.07 deg/s for 0.7 Hz, and <0.1 deg/s for 0.15 Hz).

### Modeling direction discrimination thresholds

In our previous work, a framework for predicting direction discrimination thresholds for translational motions was introduced. The same approach was used for the present paper, but here we consider a transfer function specific for the semi-circular canals.

**Fig. 4:**
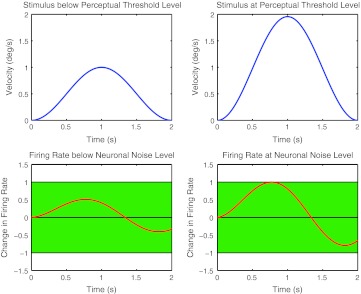
The *upper row* shows the motion stimulus, and the *lower row* the change in firing rate due to the stimulation. The *shaded area* represents the neuronal noise level. The modeling idea is based on the following assumption: In order for a stimulus to be correctly discriminated (in 75 % of the trials), the change in firing rate must be at the neuronal noise level (*right column*) 223 × 180 mm (300 × 300 DPI)

The underlying idea of the model is that, in order to be able to correctly perceive the direction of motion, the firing rate of a semi-circular canal neuron has to change from resting firing rate by an amount larger than the inherent noise of the firing rate. The relation between an inertial motion stimulus and change in firing rate of a semi-circular canal neuron can be mathematically described with a linear differential equation or, equivalently, with a transfer function (Fernandez and Goldberg 1971). Given such a transfer function, the threshold for a velocity profile, in terms of its peak velocity, can be found by scaling the peak velocity such that the change in firing rate is just at the noise level (Fig. 4). The noise level can be arbitrarily set to a value of one, since the model includes a scaling factor *K* that scales inversely with the chosen noise level during the fitting process. This implies that the *firing rate* signal has arbitrary units; it does not represent the real firing rate, but a signal that linearly scales with the firing rate.

Note that this approach takes into account the transient of the response because it operates in the time domain as opposed to working with the inverse of the steady-state gain. For further details, we refer the reader to our previous work (Soyka et al. 2011).

### A transfer function describing the semi-circular canals

The semi-circular canals can be described with a torsion pendulum model (van Egmond et al. 1949). In order to describe the neuronal response, Fernandez and Goldberg (1971) added an adaptation term and a lead component. Previous work (Hosman and van der Vaart 1978; Hosman 1996; Heerspink et al. 2005) showed that in order to describe perceptual thresholds, the adaptation term does not necessarily have to be included. As this also reduces the number of parameters, the following transfer function structure will be used throughout this work:

2 $$ H(s) = K \cdot \frac{{s \cdot (1 + \tau_{N} s)}}{{(1 + \tau_{1} s)(1 + \tau_{2} s)}} $$

This model describes the neuronal firing rate of a semi-circular canal neuron as a function of angular velocity of the head. For a short introduction to transfer functions, see the Appendix in Soyka et al. (2011).

The original formulation of the transfer function (Hosman and van der Vaart 1978) described the neuronal response to an angular acceleration. Since here angular velocity is used as input, an additional term s in the numerator is needed to convert the input signal to acceleration.

The two terms $(1 + \tau_{1} s)$ and $(1 + \tau_{2} s)$ in the denominator of the transfer function describe the deflection of the cupula in response to an angular acceleration of the head, and depend on the moment of inertia of the endolymph and the cupula, on the viscous damping of the endolymph, and on the spring stiffness of the cupula. The term $(1 + \tau_{N} s)$ in the numerator corresponds to the lead term introduced by Fernandez and Goldberg (1971) and implies that the system is sensitive to both cupular displacement and the velocity of this displacement—corresponding to sensitivity to the time derivative of acceleration. The parameter *K* is a scaling factor (see previous section) and has the units s^2^/deg to ensure that the output of the transfer function is unitless. The parameter $\tau_{2}$ was calculated for both squirrel monkeys and humans (based on the work of Igarashi, 1967) by Fernandez and Goldberg (1971) and therefore is set to 0.005 s. This reduces the number of free parameters of the model to three: $K,\tau_{1} ,\tau_{N}$.

### Fitting the parameters of the transfer function

In order to find parameters such that the model responses optimally match the measurements, an iterative error minimization procedure was used. Based on an initial choice for the parameters (Haque et al. 2004), model responses were calculated and an error function measuring the dissimilarity between responses and measurements was evaluated. A local minimum (within a certain tolerance level) of the error function could be found by systematically varying the parameter set and re-evaluating the error function until the best-fitting set was found. The sum of squared errors (SSE) was used as an error function, and the search was implemented with a nonlinear least-squares curve fitting method (“lsqnonlin” function, MATLAB, MathWorks, MA, USA).

## Results

The measured threshold estimates were averaged across participants on a logarithmic scale. This was because threshold measurements are only normally distributed (according to a Gaussian distribution) when expressed in logarithmic units (Benson et al. 1989; Grabherr et al. 2008). However, for convenience, the thresholds are reported in deg/s (Table 1). As expected, the thresholds decrease with increasing frequency (Benson et al. 1989; Grabherr et al. 2008) and are similar between different profiles with the same period length.

**Table 1:** Direction discrimination thresholds averaged on logarithmic scale over ten participants. The standard error of the mean (SEM) is given in parentheses (±)

Condition	Commanded shape	Frequency (Hz)	Threshold ± SEM (deg/s)
I	Triangular	0.15	1.984 (0.373/0.314)
II	Sinusoidal	0.15	2.552 (0.552/0.454)
III	Trapezoidal	0.15	2.124 (0.457/0.376)
IV	Triangular	0.7	0.939 (0.111/0.099)
V	Sinusoidal	0.7	1.051 (0.112/0.101)
VI	Trapezoidal	0.7	0.897 (0.107/0.096)
VII	Triangular	3	0.804 (0.030/0.029)
VIII	Sinusoidal	3	0.778 (0.051/0.049)
IX	Trapezoidal	3	0.766 (0.038/0.036)

Note that the SEM is asymmetric because it was calculated on a logarithmic scale and that the actual profile shape for the 3-Hz profiles deviated from the commanded shape

On average, it took 90 trials per profile until the variance of the estimate dropped below the break value and the threshold estimate could be determined. The maximum number of trials needed for one profile was 149.

**Fig. 5:**
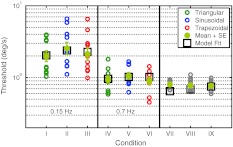
Individual threshold data for all participants. The mean is *plotted* together with the standard error and the best model fit. Note that for conditions VII, VIII, and IX, the actual profile deviated from the commanded motion. The model accurately describes the average data 112 × 70 mm (300 × 300 DPI)

The data for the individual participants are shown in Fig. 5 together with the mean thresholds for the 9 conditions and the best model fit. The fit is based on the measured yaw velocity profiles and not on the commanded motions. The parameters found from the best fit were *K* = 0.68 s^2^/deg, $\tau_{1} = 0.68s$ and $\tau_{N} = 0.030s$ (SSE = 0.107) where $\tau_{2} = 0.005s$ was fixed.

## Discussion

### Measurements and asymmetry analysis

As expected from the characteristics of semi-circular canal neurons (their firing rate being proportional to stimulus velocity; Fernandez and Goldberg 1971, Haque et al. 2004) and as suggested or assumed from perceptual threshold modeling of others (Hosman and van der Vaart 1978; Benson et al. 1989; Heerspink et al. 2005; Grabherr et al. 2008), it was found that the discrimination process mainly depends on the peak velocity of the stimulus. The profile shape does not influence the threshold (when the threshold is expressed in terms of velocity). Benson et al. (1989) inferred this property from threshold measurements for sinusoidal acceleration stimuli with varying period length. Since reciprocal velocity thresholds, which are assumed to be proportional to the gain of the perceptual system, only slightly changed with period length, they concluded that the system is sensitive to velocity.^3^ This inference, based on only sinusoidal stimuli, assumes that the perceptual system behaves linearly. In this work, for the first time, it was shown that the discrimination thresholds in terms of velocity are actually similar for all profile shapes^4^, further supporting the conclusion that the system is sensitive to velocity.

In comparison with the results from our previous study (Soyka et al. 2011), there are striking differences between translational and rotational thresholds. For translational movements, a combination of the acceleration and the jerk (the time derivative of acceleration) of the motion determined the threshold, whereas for rotational motions, peak velocity is mainly important. This is in agreement with conclusions that Benson et al. (1986, 1989) drew based on their measurements and the linearity assumption.

As explained in the methods, it was assumed that there are no differences in discrimination performance between leftward and rightward rotational stimuli. In order to verify this assumption, the trials of the stimuli levels next to the individual thresholds of each participant were collected and combined over all conditions yielding on average 155 trials per direction and participant. Pearson’s chi-square test (2-sided) was used to assess whether the performance depends on the direction of the rotation (Table 2). No significant performance differences between leftward and rightward rotations were found in this post hoc analysis justifying the performed fitting approach. Note that the present study was not designed to search for asymmetries in perception. However, Roditi and Crane (2012) who specifically assessed this question found perceptual asymmetries differing between participants and conditions.

**Table 2:** Pearson’s chi-square test (2-sided) was used to assess whether the performance depends on the direction of the rotation

Participant	1	2	3	4	5	6	7	8	9	10
*p* value	.317	.679	.591	.724	.686	.170	.961	.559	.379	.186

For each participant, the trials of the stimuli levels next to the individual thresholds of each participant were combined over all conditions yielding on average 155 trials per direction. No significant performance differences between leftward and rightward rotations were found

### Model fit

The proposed model is able to accurately fit the measurements. It correctly describes the decrease in thresholds with increasing frequency and the similarity of thresholds for different profiles with the same frequency. Note that the thresholds are only similar between different profiles when expressed in the canonical units of velocity. If expressed in terms of peak acceleration, the thresholds actually differ: Triangular profiles require higher accelerations compared to trapezoidal profiles to reach the same peak velocity for a fixed period length (Fig. 1).

Comparing the best parameter estimates to other literature is difficult, since there are no available electrophysiological measurements of the semi-circular canal afferents for humans. Dai et al. (1999) estimated $\tau_{1} = 4. 2s$ based on angular vestibulo-ocular reflex measurements in humans and reported that monkeys exhibit a similar time constant. Theoretical predictions for $\tau_{1}$ in humans, based on the anatomy of the semi-circular canals, are around 10 s (van Egmond et al. 1949). Concerning $\tau_{N}$, there are no predictions for humans, because this time constant is related to hair cell physiology and not anatomy. Since $\tau_{1}$ was reported to be similar in humans and monkeys, and theoretical predictions for $\tau_{N}$ are not available, we resort to comparing our results to recordings from monkeys.

Haque et al. (2004) fit a comparable model to recordings from regular firing semi-circular canal afferents of rhesus monkeys and found $\tau_{1} = 4s$ and $\tau_{N} = 0.013s$. The parameters estimated from the present human data are $\tau_{1} = 0.68s$ and $\tau_{N} = 0.030s$. While $\tau_{N}$ is in a similar range to the value found in monkeys, $\tau_{1}$ is smaller. This might be due to the small number of frequencies included in the fit and will be further discussed below. In accordance with the findings of Grabherr et al. (2008), it seems that velocity storage does not affect perceptual thresholds for rotations, because the associated time constant $\tau_{1}$ is smaller than what is expected from velocity storage literature ($\tau_{vel} = 16s$, Young and Oman 1969).

### Parameter estimation based on combined data from three studies

The goals of this work were to measure different profile shapes with the same period length and to demonstrate that the proposed modeling approach is able to accurately describe the data. This has been successfully shown and the next step would be to measure thresholds over a larger frequency range to get a better estimate of the parameters of the underlying transfer function. Since our thresholds were measured for similar motions and are comparable in magnitude to the thresholds measured by Benson et al. (1989) and Grabherr et al. (2008), an attempt was made to combine the three data sets and to find the best model parameters for this large data set. The results from this combined data set should be interpreted with caution, because of the differences between the setups used to gather the data and the slight differences in the definition of threshold. Benson et al. (1989), and our study defined the threshold as the stimulus intensity yielding 75 % correct discrimination performance, whereas Grabherr et al. (2008) used a 3-down, 1-up staircase paradigm targeting at 79.4 % correct discrimination performance.

**Fig. 6:**
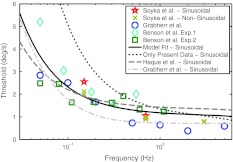
Combined data set of Benson’s, Grabherr’s and the present data. The model fit *lines* represent the predictions for sinusoidal acceleration profiles 127 × 88 mm (300 × 300 DPI)

The best model fit to the combined data set, consisting of 30 data points, was calculated and the estimated model parameters are* K* = 2.04* s* ^2^/deg, $\tau_{1} = 2.16s$, and $\tau_{N} = 0.014s$ (SEE = 8.633). The combined data set together with threshold predictions for sinusoidal accelerations from different models is shown in Fig. 6.

The solid line represents the fit of our model to the combined data set. The dotted line shows the model predictions based on the parameters found by fitting the model only to the data gathered in this study. The dashed line illustrates the predictions of our model using the parameter estimates^5^ from Haque et al. (2004), and the dash-dotted line depicts the model of Grabherr et al. (2008).

It can be seen that all models correctly describe the qualitative behavior of the measurements: Thresholds increase with decreasing stimulus frequency. Since the model underlying the dotted line is based on the measured yaw velocity profiles (including noise), the threshold predictions for ideal (noise free) stimuli are above the actually measured thresholds. The parameter estimates for $\tau_{1}$ range from $\tau_{1} = 0.68s$ (only present data), $\tau_{1} = 0.70s$ (Grabherr et al. 2008), $\tau_{1} = 2.16s$ (combined data), to $\tau_{1} = 4s$ (Haque et al. 2004); all of which are smaller than $\tau_{vel} = 16s$, as one would expect to find if velocity storage influenced the thresholds. Grabherr et al. (2008) suggested a shortening of $\tau_{1}$ as compared to what one would expect to find if the perceptual dynamics were governed by the dynamics of the peripheral sensory system. The identified time constant using only the present data is in accordance with the findings of Grabherr et al. (2008). However, the time constant based on the combined data set is in between $\tau_{1} = 0.70s$ and $\tau_{1} = 4s$ as suggested by data from the peripheral sensory system (Haque et al. 2004) or $\tau_{1} = 4. 2s$ as suggested by angular vestibulo-ocular reflex measurements in humans (Dai et al. 1999). Although $\tau_{1} = 2.16s$ (combined data set) is still shorter than $\tau_{1} = 4s$, this estimate is closer to the sensory dynamics than previous findings. More measurements, especially for low-frequency stimuli, are needed to assess whether there really is a shortening of $\tau_{1}$ or whether the dynamics of perceptual thresholds are governed by the dynamics of the semi-circular canals. The estimated value $\,\tau_{N} = 0.014s$ for the combined data set is in close agreement with the estimate $\tau_{N} = 0.013s$ from Haque et al. (2004). These findings add to the discussion of whether vestibular ocular motor reflexes, as opposed to voluntary saccadic eye movements, are processed differently than perceptual information (Van Beuzekom and Van Gisbergen 2000; Merfeld et al. 2005a, b; Barnett-Cowan et al. 2005; Park et al. 2006; Barnett-Cowan and Harris 2008; Bertolini et al. 2011).

Both modeling approaches, the one used by Grabherr et al. (2008) and ours, are able to accurately describe thresholds for sinusoidal motion profiles. Our approach has the advantage that it can also take into account non-sinusoidal motion profiles and that the structure of the transfer function is similar to transfer functions used in studies describing the physiology of the semi-circular canals. Therefore, it is easier to directly compare the parameters gained from perceptual measurements to parameters found from physiological measurements.

## Conclusions

In this work, threshold measurements for discriminating the direction of yaw rotations were presented. The period length of the acceleration and the shape of the acceleration profile were varied. In agreement with theoretical predictions, it was found that the peak velocity of the profile was the determining factor for discrimination. We also introduced a model that can take both period length and profile shape into account. The model is based on a transfer function describing the firing rate of semi-circular canal neurons responding to inertial motion. The estimated model parameters, in agreement with previous findings, suggest that velocity storage does not influence the dynamics of perceptual thresholds.

The parameter estimates from the combined data set are similar to findings from electrophysiological recordings in monkeys, supporting the hypothesis that the dynamics of perceptual thresholds are governed by the dynamics of the peripheral sensors. However, estimates based on only our data suggest a shortening of the time constant $\tau_{1}$ as compared to the sensory dynamics. Further research is needed to decide whether there is a shortening of $\tau_{1}$.

The proposed model is important as it links physiologically meaningful parameters to perceptual measurements. This link might in turn facilitate the assessment of vestibular disorders from perceptual measurements. Perception-based vestibular diagnostics have recently been discussed by Merfeld et al. (2010) and might, for example, prove helpful in patients who, in addition to having vestibular problems, also have ocular motility problems which confound standard eye movement–based diagnostics.

## Footnotes

1 The terms “period length” and “frequency” are used interchangeably, and the frequency *f* in Hertz is defined as the inverse of the period length *T* in seconds: *f* = 1/*T*.

2 Please note that Benson et al. (1989) reported the thresholds in terms of peak velocity *A*_vel_. For better comparability, we refer to the behavior in terms of peak acceleration *A*_acc_. The two are related through the period length *T* of the stimulus: $A_{\text{vel}} \propto A_{\text{acc}} \cdot T$.

3 For further explanation of this reasoning, we refer the reader to the Appendix of Soyka et al. 2011.

4 Note that the 3-Hz profiles were not correctly reproduced and therefore were not as distinct in their shape as the profiles for the other frequencies.

5
$\tau_{1} = 4s$, $\tau_{2} = 0.005s$, $\tau_{N} = 0.013s$, and * K* = 2.97 *s* ^2^/deg, where *K* was adjusted such that the data set was fit best.
